# Determining health-related quality of life among children with malaria in Kenya: does the choice of EQ-5D value set matter?

**DOI:** 10.1186/s12955-025-02445-0

**Published:** 2025-11-03

**Authors:** Tabitha Okech, Montarat Thavorncharoensap, Usa Chaikledkaew, George Paul Omondi, Patricia Nyokabi, S. M. Thumbi, Ammarin Thakkinstian

**Affiliations:** 1 Mahidol University Health Technology Assessment (MUHTA) Graduate Program, Bangkok, Thailand; 2 Center for Epidemiological Modelling and Analysis, University of Nairobi, Nairobi, Kenya; 3 Department of Pharmacy, Faculty of Pharmacy, Mahidol University, Bangkok, Thailand; 4 Animal Health Innovation Lab, Department of Clinical Studies, University of Nairobi, Nairobi, Kenya; 5 Ministry of Health, Nairobi, Kenya; 6 Paul G. Allen School for Global Health, Washington State University, Washington, USA; 7 Institute of Immunology and Infection Research, University of Edinburgh, Edinburgh, UK; 8 Department of Clinical Epidemiology and Biostatistics, Faculty of Medicine Ramathibodi Hospital, Mahidol University, Bangkok, Thailand

**Keywords:** EQ-5D, Utility, Malaria, Quality of life, Value set, Kenya

## Abstract

**Purpose:**

Malaria remains a major public health issue, especially for children under five, yet health-related quality of life (HRQoL) data needed for cost-effectiveness analyses are limited. This study assesses malaria’s impact in Kenyan children and examine how different EQ-5D value sets affect utility estimates.

**Methods:**

A cross-sectional study was conducted in western Kenya. From June to September 2024, caregivers completed the proxy-reported EQ-5D-5L questionnaire. In the absence of a Kenyan value set, utility scores were estimated using both Ethiopian, Ugandan, and Dutch value sets. Differences across value sets were assessed using the Friedman test, while factors associated with utility scores were identified using Tobit regression.

**Results:**

Of the 95 children studied, 45 had uncomplicated malaria and 50 had severe malaria. The most affected EQ-5D-5L domains were pain/discomfort and usual activity. For uncomplicated malaria, the median (interquartile) utility scores were 0.82 (0.86) using the Ethiopian value set, 0.59 (1.29) using the Ugandan value set, and 0.73 (0.98) using the Dutch value set. For severe malaria, the median (interquartile) utility scores were lower: 0.30 (1.21) for the Ethiopian, -0.13 (1.44) for the Ugandan, and 0.09 (0.96) for the Dutch value sets, respectively. The differences across these value sets were statistically significant. Vaccination status and severity of malaria were found to be significant determinants of utility scores.

**Conclusion:**

Malaria considerably reduces children’s HRQoL. The choice of value set has a significant influence on utility estimates, potentially affecting the cost-effectiveness analyses of malaria interventions. These findings underscore the need for Kenyan-specific value set to reflect preference of the population.

## Introduction

Malaria continues to pose a significant public health threat, particularly in sub-Saharan Africa. According to the World Malaria Report, there were 263 million cases of malaria and 597,000 related deaths in 2023 [1]. Notably approximately 95% of these deaths occurred in Africa [1]. Children under the age of five were the most affected, accounting for 76% of the total fatalities [2]. In 2019, malaria was identified as the leading pathogenic cause of Disability-Adjusted Life Years (DALYs) among children under five [3]. According to the Global Burden of Disease study, malaria was ranked as the fifth leading cause of DALYs for children aged 0–9 [4].

Malaria not only contributes to mortality but also significantly impacts quality of life. Symptoms such as fever, muscle pain, fatigue, and headaches [5] affect physical health and can disrupt daily activities, leading to reduced productivity. In young children, especially those under 5 years old, malaria impairs cognitive and learning ability and causes low birth weight, mobility and neural impairment [6]. In addition, the disease places a financial burden on individuals and families due to treatment expenses, lost work or school days, and long-term health effects, particularly in children [6]. Repeated infections can hinder growth, impair cognitive development, and disrupt education [6].

Measuring the health-related quality of life (HRQoL) in malaria is crucial because it helps capture the full impact of the disease. Furthermore, measuring and valuing HRQoL in terms of utility is an essential part of economic evaluation, allowing the estimation of quality-adjusted life-years (QALYs) to compare outcomes and value for money across health interventions. Given malaria’s significant health and economic burden, a cost-effectiveness study of malaria intervention is crucial to optimizing resource allocation for malaria control, contributing to sustainable, evidence-based malaria control efforts that can improve public health outcomes and reduce the disease’s economic impact.

Currently, there is a lack of sufficient data on HRQoL, measured in terms of utility for malaria to support cost-effectiveness analyses. The most investigated HRQoL outcomes associated with malaria were DALYs [7]. Due to the limited availability of utility values, most cost-effectiveness studies have used DALYs as the outcome measure instead of QALYs [8]. Nevertheless, it should be noted that DALY and QALY cannot be used interchangeably [9]. To date, only four studies that assessed utility values in malaria patients have been identified [7]. Although children are the most affected group, none of the studies focused on utility values among children [10–13]. Two studies investigated utility values among patients with uncomplicated malaria [11, 12], while another reported utility values regardless of malaria severity [10]. Of the four studies, two each adopted EQ-5D-5L [11, 13] and EQ-5D-3 L [10, 12].

Malaria continues to be a significant public health issue in Kenya, with almost 70% of the population at risk [14]. Each year, about 4,000 malaria-related deaths are reported in the country [15]. It is also the leading cause of death among children under the age of five [16, 17], causing 20–30% mortality among children, with cascading direct and indirect effects on the quality of life among those affected and their carers [18, 19]. In Kenya, health technology assessment (HTA) is being increasingly integrated into the health system as part of the move toward Universal Health Coverage (UHC). Kenya’s commitment to achieve UHC has driven the establishment of organization structures for implementation and institutionalization of HTA. In 2018, the Ministry of Health in Kenya implemented a reform aimed at establishing a transparent process for setting healthcare priorities in the development of the national health benefits package, which included a mandate to create a framework for the institutionalization of HTA in the country [20, 21]. With malaria being a leading cause of morbidity and mortality, cost-effectiveness analysis is warranted in Kenya to help ensure that available resources are allocated efficiently to interventions that provide the most significant health benefits.

This study addresses critical gaps in the literature examining HRQoL, measured as utility values, among Kenyan children under five years of age with malaria, stratified by the severity of malaria. In accordance with the EuroQol Group guidance [22, 23], as no EQ-5D-5L value set exists for Kenya, we compared utility values estimated from Ethiopia [24] and Uganda [25]—countries with comparable socio-demographic and regional characteristics—to evaluate the effect of value set choice. Furthermore, the Netherlands value set [26] was also included, as the Netherlands was one of the original developers of the EQ-5D-5L instrument. Additionally, we identify key factors associated with utility scores. Thus, this study contributes valuable data for future economic evaluations of malaria interventions, enhancing the applicability of HTA evidence in informing health policy decisions in the Kenya and similar settings.

## Methodology

A cross-sectional study involving multiple centers was carried out. Kenya is classified into five malaria zones with varied risks: coastal endemic, lake endemic, seasonal malaria transmission, malaria epidemic-prone areas of western highlands and epidemic-prone areas, and low-risk malaria areas [27]. The malaria vaccine pilot implementation was conducted in Kenya’s lake endemic zone, which comprises eight counties. Although the lake zone is generally considered a high-risk area for malaria, transmission intensity can vary within it. Therefore, the eight counties selected for the pilot were further categorized into high-, moderate-, and low-transmission areas using a multi-stage selection method. In the second stage, one county was randomly chosen within each transmission zone. In the third stage, 2 health facilities (i.e., one health center and one hospital) were randomly selected in each county.

This study recruited participants from six health facilities in the selected three counties (i.e., Busia, Kakamega, and Homabay) located in the lake endemic zone. The prevalence of malaria in these counties range from 8% to 37% [28], with favorable eco-epidemiological attributes for malaria occurrence and transmission [14], facilitating year-round transmission and resulting in higher infection levels among children and pregnant women [14]. The eligibility criteria were as follows: (1) children diagnosed with malaria, (2) aged less than 5 years old, (3) living in the selected three counties, (4) availability of caregiver who closely took care of the child and can communicate in Dholuo, Swahili, or English, and (5) willing to participate in the study. Children with known co-morbidities (i.e., diagnosed with other acute/chronic illnesses) were excluded.

### Sample size

The sample sizes required to estimate the utility values for uncomplicated malaria and severe malaria were calculated from the following formula [29]:

$$\:n={\frac{{Z}_{\alpha\:/2}}{({\mu\:\varepsilon\:)}^{2}}}^{2}{\sigma\:}^{2}$$

Where n was the required sample size; α referred to the significance level or the probability of making a type I error; σ^2^ was the variance of utility value; µ was the expected mean utility value; and 𝜺 was the margin of error. The sample size was calculated separately for children with uncomplicated malaria and with severe malaria. For uncomplicated malaria, with the type I error of 0.05, σ^2^ of 0.038025, the mean utility of 0.571 [13], and 10% margin of error, the required sample size was 45. On the other hand, with the σ^2^ of 0.015876, the mean utility of 0.349 [13], and 10% margin of error, the sample size required for severe malaria was 50.

### Data collection

Data collection was conducted from June through to September 2024 at the following six facilities in 3 selected counties: Kakamega County Referral Hospital, Elwesero Health Centre, Matungulu Health Center, Busia County Referral Hospital, Homa Bay County Referral Hospital, and Makongeni Health Center. All children who received a laboratory-confirmed malaria diagnosis and met the eligibility criteria were invited to participate in the interview. Our study defined severe malaria as a laboratory-confirmed infection with clinical complications requiring hospitalization [30].

A face-to-face interview was conducted by trained interviewers using a structured questionnaire. In the first part of the questionnaire, general and clinical characteristics, including age, gender, type of proxy, severity of malaria, and malaria vaccination status, were examined. The second component assessed health utility through the proxy version of the EQ-5D-5L instrument, administered in either English or Swahili [31]. The EQ-5D questionnaire is the most recommended measure by national HTA agencies as the choice of multi-attribute utility measure (MAUM) for cost-utility analysis [32]. Besides the lack of consensus on the instrument used to measure utility among children, a previous systematic review indicated that EQ-5D was the most commonly used generic utility measure in children and adolescents [33]. In addition, the EQ-5D was the most commonly used preference-based measure for malaria, and the EQ-5D-5L demonstrated robust psychometric properties in patients with uncomplicated malaria [11].

EQ-5D-5L was recommended for self-completion by individuals aged 16 years and older [34]. It comprises the EQ-5D descriptive system and EQ visual analogue scale (EQ-VAS). The EQ-5D descriptive system consists of five dimensions: mobility, self-care, usual activities, pain/discomfort, and anxiety/depression. For EQ-5D-5L, each dimension has five levels ranging from 1 (no problem) to 5 (extreme problem or unable to function). On the other hand, EQ-VAS is a vertical visual analogue scale used to measure an individual’s assessment of their current HRQoL. The ratings were categorized as “The best health you can imagine”- scored 100, and “The worst health you can imagine”- scored zero [35, 36]. Measuring HRQoL among very young children (under five) is methodologically and conceptually challenging; hence, using a proxy is an alternative and common approach [33]. For patients with uncomplicated malaria, we interviewed the proxies during outpatient visits following the malaria diagnosis. In the case of severe malaria, the proxies were interviewed within 24 h of the child’s admission to the inpatient department. In our study, the EQ-5D-5L Proxy Version 2 was used, whereby a caregiver familiar with the patient provides responses based on how they believe the patient would rate their own health [23].

This study received authority transfer from Mahidol University Institutional Review Board-MU-DT/PY-IRB to Maseno University Scientific and Ethics Review Committee, Kenya. It was approved by Maseno University (MUSERC/01321/23) and the Kenya National Council for Science, Technology and Innovation (NACOSTI/P/24/35525). Each participant’s parent or legal guardian provided written informed consent before data collection. The EuroQol group granted permission to use the EQ-5D-5L questionnaire.

### Data analysis

Demographic data and clinical characteristics of the participants were summarized in terms of mean (standard deviation, SD), median (interquartile range), and frequency (percentage). The rate of patients who reported having problems in each EQ-5D-5L dimension was reported. The dimension with the highest proportion of respondents reporting “severe” or “extreme problems” (level 4 or 5) was considered the most affected dimension [31]. The negative utility value represents a health state worse than a death health state; 0 represents death, and 1 represents full health [37].

In the absence of locally developed value sets, it is recommended to adopt value sets from countries with similar populations, taking into account socio-demographic, cultural, and linguistic factors that are likely to affect health preferences [22, 23]. Due to the unavailability of Kenya’s specific value set, each EQ-5D health state was, therefore, converted to health utility value using Ethiopia [24] and Uganda [25] value sets. The value sets from Uganda and Ethiopia were adopted, as they are the only two currently available in Africa. As EQ-5D was developed by the EuroQol group- a collaboration among researcher from 5 European countries: the Netherlands, the United Kingdom, Sweden, Finland, and Norway [38]- and since the Netherlands continues to be a major center for EQ-5D-related research [38], the Dutch value set [26] was also employed.

The three value sets were developed using the EuroQol Valuation Technology (EQ-VT) protocol. For Ethiopia and the Netherlands, the protocol combined time trade-off and discrete choice methods, while Uganda included only time trade-off method. The valuation studies were conducted with nationally representative adult samples: 545 in Uganda, 1,050 in Ethiopia, and 1,003 in the Netherlands to reflect general population preferences. The value sets for EQ-5D-5L health state ranged from − 0.718 to 1 for the Ethiopia value set, -1.116 to 1 for the Uganda value set, and − 0.446 to 1 for Dutch value set. In the Ethiopian and Dutch value sets, anxiety/depression emerged as the most important dimension, whereas for Uganda, pain/discomfort held the greatest importance. Conversely, self-care was considered the least important dimension in both the Ethiopian and Dutch value sets, while anxiety/depression was ranked lowest in the Ugandan set. The second-best health states identified were 12,111 (0.976) for Ethiopia, 11,112 (0.950) for Uganda, and 21,111 (0.918) for the Netherlands.

Comparisons of categorical variables between patients with uncomplicated and severe malaria were conducted using the Chi-square test or Fisher’s exact test when expected cell counts were low. Due to the non-normal distribution of the data, the Mann–Whitney U test was employed to compare continuous variables and utility values between the two patient groups. The Friedman test was utilized to assess differences in utility scores across the three value sets. When a significant result was observed, pairwise comparisons were conducted using the Wilcoxon signed-rank test as a post-hoc analysis. Tobit regression was used to ensure that predictions respect the natural bounds of HRQoL utilities, which are typically bounded between 0 (or below 0 in some cases for states worse than death) and 1 [37]. In our study, multivariate Tobit regression analysis was, therefore, adopted to examine factors predicting health utility among malaria patients. Guided by the underlying theoretical/conceptual framework, variables that are known or hypothesized to influence the utility values and demonstrated significant association with utility scores in univariate tobit regression were included in the multivariate tobit analysis. All statistical tests were two-tailed, and significance was set at *p* < 0.05 except for univariate analysis in which a p value < 0.2 was used to determine candidate factors for multivariate analysis.

## Results

A total of 45 and 50 children with uncomplicated malaria and severe malaria were included in the study, of whom 39/95 (41.1%) were male, and 56 (58.9%) were female. There was no significant difference in terms of age of patients with uncomplicated malaria (25.4 months ± 13.36) and severe malaria (28.42 months ± 18.71), as shown in Table 1. Similarly, no significant difference in terms of the age of proxies was reported. As shown in Table 2, there was no significant difference between uncomplicated and severe malaria in terms of general characteristics except for the gender of patients and the education of proxies. Proxies in the severe malaria group reported more individuals with education beyond secondary school.

**Table 1:** Age of patients and proxies

Variable	Uncomplicated malaria		Severe malaria		*P*-Value
	*n*	Mean (SD) / Median (IQR)	*n*	Mean (SD) / Median (IQR)	
Age of children (months)	45	25.4 (13.36)/ 24 (22)	50	28.42 (18.71)/ 27.5 (36)	0.4532
Age of proxy (Years)	45	30.67 (11.95)/ 26 (6)	50	29.98 (9.97)/ 28 (9)	0.7199

**Table 2:** General characteristics of patients and proxies

		Uncomplicated malaria		Severe malaria		*P*-Value
		*N*	(%)	*N*	(%)	
Gender of children						< 0.001
	Female	18	40.00%	38	76.00%	
	Male	27	60.00%	12	24.00%	
Gender of proxy						0.185
	Male	2	4.44%	6	12.00%	
	Female	43	95.56%	44	88.00%	
Type of proxy						0.007
	Father	0	0%	9	18.00%	
	Mother	40	100%	39	78.00%	
	Other	0	0%	2	4.00%	
Education of proxy						0.007
	No education	4	8.89%	0	0%	
	Primary school	23	51.11%	22	44.00%	
	Secondary school	17	37.78%	17	34.00%	
	Higher than secondary school	1	2.22%	11	22.00%	
Marital status						0.833
	Married	39	86.67%	45	90.00%	
	Never married	3	6.67%	3	6.00%	
	Divorced/separated/widow	3	6.67%	2	4.00%	
Occupation						0.126
	Unemployed	32	71.11%	28	56.00%	
	Informally employed	13	28.89%	19	38.00%	
	Formally employed	0	0%	3	6.00%	
Child’s residence						0.740
	Rural	31	68.89%	36	72.00%	
	Urban	14	31.11%	14	28.00%	
Child received malaria vaccine						
	No	25	55.56%	29	58.00%	0.810
	Yes	20	44.44%	21	42.00%	

The distribution of responses to each dimension of the EQ-5D-5L was reported in Table 3. There were significant differences in the distribution of pain/discomfort and anxiety depression domains when comparing uncomplicated malaria with severe malaria. The findings indicated that patients with severe malaria reported a higher proportion of severe to extreme problems for pain/discomfort and anxiety/depression domains, as compared to patients with uncomplicated malaria. The most affected dimensions of EQ-5D-5L among severe malaria were pain/discomfort, followed by anxiety, depression, and usual activities. On the other hand, the most affected dimension of EQ-5D-5L among uncomplicated malaria was pain/discomfort, followed by self-care, and usual activities.

**Table 3:** Frequency distribution of responses to each dimension of the EQ-5D-5L

Dimension	Level	Uncomplicated malaria (%)	Severe malaria (%)	*P*-value
Mobility	No problem	14 (31.1%)	12 (24.0%)	0.461
	Slight problem	10 (22.2%)	7 (14.0%)	
	Moderate problem	9 (20.0%)	9 (18.0%)	
	Severe problem	6 (13.3%)	9 (18.0%)	
	Extreme problem	6 (13.3%)	13 (26.0%)	
Self-care	No problem	14 (31.1%)	9 (18.0%)	0.159
	Slight problem	10 (22.2%)	10 (20.0%)	
	Moderate problem	8 (17.8%)	5 (10.0%)	
	Severe problem	8 (17.8%)	12 (24.0%)	
	Extreme problem	5 (11.1%)	14 (28.0%)	
Usual activities	No problem	11 (24.4%)	9 (18.0%)	0.071
	Slight problem	14 (31.1%)	8 (16.0%)	
	Moderate problem	8 (17.8%)	6 (12.0%)	
	Severe problem	8 (17.8%)	13 (26.0%)	
	Extreme problem	4 (8.9%)	14 (28.0%)	
Pain/Discomfort	No problem	3 (6.7%)	2 (4.0%)	0.001*
	Slight problem	21 (46.7%)	5 (10.0%)	
	Moderate problem	7 (15.6%)	12 (24.0%)	
	Severe problem	8 (17.8%)	19 (38.0%)	
	Extreme problem	6 (13.3%)	12 (24.0%)	
Anxiety/Depression	No problem	13 (28.9%)	7 (14.0%)	0.002*
	Slight problem	15 (33.3%)	4 (8.0%)	
	Moderate problem	6 (13.3%)	10 (20.0%)	
	Severe problem	7 (15.6%)	16 (32.0%)	
	Extreme problem	4 (8.9%)	13 (26.0%)	

Table 4 shows the mean (SD) and median (IQR) utility values for uncomplicated and severe malaria using the Ethiopian and Ugandan value sets. For uncomplicated malaria, the mean (SD) utility value using the Ethiopian value set, Ugandan value set, and the Dutch value set was 0.54 (0.54), 0.29 (0.69), and 0.49 (0.49), respectively. For children with severe malaria, the Ethiopian value set yielded a mean (SD) utility of 0.17 (0.61), while the Ugandan value set yielded a mean (SD) of -0.16 (0.71), and the Dutch value sets yield a mean (SD) of 0.16 (0.48) The median utility ranged from 0.59 to 0.82 for uncomplicated malaria and from − 0.13 to 0.30 for severe malaria, respectively. The VAS scores showed a mean (SD) of 61.40 (13.45) for uncomplicated malaria and 58.26 (12.06) for severe malaria. The findings indicated that severe malaria patients reported significantly lower utility values than uncomplicated malaria patients, regardless of the types of value sets. For each severity level, there was also a significant difference in utility values derived from different value sets, with significantly lower values reported when using the Uganda value set. No statistically significant difference in utility scores for severe malaria was found between the Ethiopia and Netherlands value sets.

**Table 4:** Utility values by severity and types of value sets

Severity		Mean (SD) / Median (IQR)			*P*-value*
	Utility (Ethiopia value set)	Utility (Uganda value set)	Utility (Dutch value set)	VAS	
Uncomplicated (*n* = 45)	0.54 (0.54) / 0.82 (0.86)	0.29 (0.69) / 0.59 (1.29)	0.49 (0.49)/ 0.73 (0.92)	61.40 (13.45) / 60.00 (19.00)	< 0.001^a^
Severe (*n* = 50)	0 0.17 (0.61) / 0.30 (1.21)	-0.16 (0.71) / -0.13(1.44)	0.16 (0.48)/ 0.09 (0.92)	58.26 (12.06) / 59.00 (16.00)	< 0.001^b^
P-value **	< 0.001	0.002	0.002	0.210	

*Friedman test comparing utility scores across 3 value sets

** Mann-Whitney Test comparing utility scores between uncomplicated and severe malaria cases

a = statistically significant differences were observed among all three value sets: Uganda vs. Ethiopia, Uganda vs. the Netherlands, and Ethiopia vs. the Netherlands, based on Wilcoxon signed-rank tests

b = significant differences were found between the Uganda and Ethiopia value sets, and between Uganda and the Netherlands, based on Wilcoxon signed-rank test

In univariate analysis, the following variables were statistically significant in affecting the utility values; proxy age, child’s gender, proxy type, educational attainment, occupation, marital status, vaccination status, malaria severity, and the number of vaccine doses received. However, a high degree of collinearity was observed between education and occupation, as well as between vaccination status and number of vaccine doses. As a result, occupation and vaccine dose were excluded from the multivariate analysis to avoid multicollinearity. Factors affecting EQ-5D-5L health utility scores using multivariate tobit analysis were reported in Table 5. For all three value sets, those with severe malaria have significantly lower utility values than those with uncomplicated malaria. (β = -0.4872, *p* < 0.001 for Ethiopia value set; β= -0.5913, *p* < 0.001 for Uganda value set; and β = -0.4256, *p* < 0.001 for Dutch value set). In addition, it was found that the children who received malaria vaccine reported significantly higher utility values compared to those who were not vaccinated (β = 0.2775, *p* = 0.013 for Ethiopia value set; β = 0.3362, *p* = 0.013 for Uganda value set; and β = 0.2314, *p* = 0.014 for Dutch value set).

**Table 5:** Factors associated with utility scores using Ethiopian and Ugandan EQ-5D-5L value sets

Variable		Ethiopia value set		Uganda value set		Dutch value set	
		β	*P* value	β	*P* value	Β	*P* value
Age of proxy		0.0025	0.71	0.0044	0.587	0.0036	0.526
Gender of Children							
	Female	Ref		Ref		Ref	
	Male	0.0618	0.614	0.7723	0.602	0.0475	0.644
Type of proxy							
	Mother	Ref		Ref		Ref	
	Father	0.3393	0.129	0.4035	0.136	0.2389	0.202
	Other	-0.0337	0.905	-0.1173	0.732	-0.1143	0.631
Education of proxy							
	No education	Ref		Ref		Ref	
	Primary school	-0.1671	0.948	0.0688	0.891	0.0029	0.993
	Secondary school	0.2604	0.537	0.3158	0.536	0.1566	0.658
	Higher education	0.5412	0.207	0.6654	0.200	0.4339	0.228
Marital status							
	Married	Ref		Ref		Ref	
	Never married	-0.2422	0.917	0.0175	0.951	0.0102	0.959
	Divorced/separated/widow	0.3297	0.321	0.4296	0.285	0.32	0.32
Malaria severity							
	Uncomplicated malaria	Ref		Ref		Ref	
	Severe Malaria	-0.4872	< 0.0001*	-0.5913	< 0.0001*	-0.4256	< 0.0001*
Malaria vaccination							
	No	Ref		Ref		Ref	
	Yes	0.2775	0.013*	0.3362	0.013*	0.2314	0.014*

*p < 0.05

## Discussions

This study assessed the impacts of malaria on HRQoL in Kenyan children under five and examined how the choice of EQ-5D-5L value set influenced utility score estimation. The results showed that malaria, particularly in its severe form, significantly compromised HRQoL. Based on our findings, the mean utility values associated with severe malaria, ranging from − 0.16 to 0.17, were notably lower than those reported for both rotavirus diarrhea—ranging from 0.593 [39] to 0.74 [40]—and influenza, which ranged from 0.62 [41] to 0.818 [42]. Similarly, the mean utility values for uncomplicated malaria, which ranged from 0.29 to 0.54 fell below the utility estimates observed for both rotavirus diarrhea and influenza. While our finding suggested that even uncomplicated malaria has a greater negative impact on HRQoL than these other common infectious diseases, the results should be interpreted with caution as direct comparisons may not be appropriate due to differences in study populations and settings.

Our study reported that pain/discomfort was the most affected dimension for severe malaria, followed by anxiety/depression and usual activities, respectively. In contrast, pain/discomfort, self-care, and usual activities were reported as the most affected dimensions for uncomplicated malaria, albeit to a lesser severity as a problem. Our findings were generally consistent with the previous studies [7], which found that pain/discomfort and usual activities were the most affected dimensions. This is possibly because common symptoms of malaria include muscle pain, fever, fatigue, and weakness [43], which could cause significant pain and discomfort, make it difficult to perform daily activities, and could also cause anxiety.

Our findings revealed significant differences in utility values derived from Uganda and Ethiopian value sets, as well as between the Ugandan and the Dutch value sets. Importantly, the magnitude of the difference seemed to be larger than that of the minimal clinically important difference (MCID) of 0.11 for EQ-5D-5L [44]. Such a significant difference raises some concerns and is likely to affect the findings of the cost-utility analysis. Without a country-specific value set, the value set from other countries that share similar socio-economic, healthcare system, and cultural characteristics could be adopted. Given the substantial cultural and economic differences between the Netherlands and Kenya, the Dutch value set may not be appropriate for application in the Kenyan context. However, it is noteworthy that the utility values for malaria patients derived from the Dutch value set were more closely aligned with those obtained from the Ethiopian value set than with those from Uganda. While both Ethiopia and Uganda are located in East Africa, Uganda appears to share greater similarities with Kenya than with Ethiopia, particularly in cultural dimensions such as ethnicity, religion, and language [45]. Conversely, Kenya aligns more closely with Ethiopia than with Uganda when considering economic development indicators [46]. It is important to acknowledge that the utility values obtained from Uganda were comparatively lower, which may be attributed to the valuation study being conducted during the COVID-19 pandemic [25]. Additionally, the anxiety/depression dimension, identified as the least significant in Uganda, was rated as the most important in both Ethiopia and the Netherlands. This difference may reflect cultural attitudes in Uganda, where mental health issues are often stigmatized and perceived as a sign of personal weakness [25]. It should also be noted that the valuation study in Uganda employed of the EQ-VT Lite protocol, which relied solely on the composite time trade-off (cTTO) method and was conducted with a relatively limited sample size. Our findings reinforce the need for a Kenyan-specific EQ-5D-5L value set.

Compared to the previous study that used EQ-5D-5L [13], which found that the utility values for uncomplicated malaria and severe malaria for Indonesians were 0.57 and 0.349, respectively, our values seemed lower. In addition, our values were lower than 0.74 for uncomplicated malaria reported in Nigeria using EQ-5D-5L. This could be explained by the fact that the previous study [11, 13] was conducted among the adult population, while our study was conducted among children aged under five, who are the most affected population with the highest mortality from malaria. Additionally, our study relied on proxy-reported data, while earlier studies used self-reported measures. It is well-documented that parent proxy-reports often yield lower HRQoL scores compared to child self-reports [47–49]. Moreover, one previous study [13] was conducted in Southeast Asia, in contrast to our study in Africa, where disparities in healthcare systems, economic conditions, and cultural contexts may influence health state preferences. Although another African study [11] was conducted in Nigeria, it applied a value set from Zimbabwe, and this variation in value sets could further contribute to the observed discrepancies.

As expected and consistent with the previous study [13], the utility score for severe malaria was lower than that of uncomplicated malaria, possibly due to the severity of symptoms. In addition, children receiving malaria vaccines had significantly higher utility values by both value sets, consistent with evidence that vaccination reduces malaria severity, complications, and caregiver stress [50]. Vaccinated children may experience milder malaria symptoms, fewer hospitalizations, and faster recovery, preserving their physical and cognitive functioning [51]. Additionally, caregivers of vaccinated children may perceive better health outcomes, influencing proxy-reported HRQoL [52]. This finding emphasizes the importance of malaria vaccination programs for disease prevention and mitigating long-term quality-of-life impairments [50].

There is no consensus about best-practice methods to measure utility in very young children (< 5 years), and data are often scarce [53]. Even in Australia [54] and UK [53], the use of the child-specific utility measures in submissions to the Pharmaceutical Benefits Advisory Committee (PBAC) and National Institute for Health and Care Excellence (NICE) was minimal. Most submissions used adult-specific measures and adult preferences to reflect children’s health [54], with most assessments using the adult version of the EQ-5D [55]. Therefore, we decided to use the EQ-5D-5L proxy version. Although EQ-5D-Y, a child-friendly version of EQ-5D, is suitable for self-completion by children aged 8–15 years, with the proxy version available for children aged 4–8 years [56], the main reason for not using EQ-5D-Y was that no value set was available for the African population, including Kenya. Without the local value set, we could not convert the responses from the EQ-5D-Y descriptive system into utility values that represent the local perspective. If the value set for EQ-5D-Y becomes available for Kenya, EQ-5D-Y should be the preferred measure of utility for children and adolescents in the country. Additionally, the EuroQol Group is in the process of developing EQ-TIPS [57, 58], a specialized instrument designed to assess HRQoL in very young children. Once fully developed and validated, EQ-TIPS has the potential to be widely adopted in future research and clinical settings to improve the accuracy and relevance of utility assessments in young children.

This study has several limitations. Firstly, as our patients were very young children (under five), proxies were involved in the interview. According to the previous systematic review, there was some evidence of discrepancies between the reported HRQoL given by children and those given by adults who adopted their perspectives or parents when adopting a children’s perspectives [59]. Secondly, the EQ-5D value sets used in this study may not accurately reflect children’s perceptions of health, as all three value sets were adult value set, which considered adults’ perspective to their own health. It should be noted that adults assigned systematically different values to health states when evaluating them for themselves compared to when assessing the same states for a 10-year-old child [60]. Furthermore, the value sets were adopted from other African countries (i.e., Ethiopia and Uganda) and the Netherlands, which may not reflect the preference of the Kenyan population. Importantly, using the different value sets yields significant differences in the utility values, which could affect the results of cost-utility analysis. Further studies examining the EQ-5D-5L and EQ-5D-Y value set for Kenya are highly warranted. In the interim, and in the absence of a locally derived value set, future studies should aim to conduct comprehensive psychometric evaluations across diverse patient populations to identify the most appropriate existing value set for application in the Kenyan context. Nevertheless, in the current study, we have demonstrated known-group validity by comparing utility differences between uncomplicated and severe malaria, which indicates that all three value sets are generally acceptable. Lastly, the utility values reported in the findings did not capture the long-term impact of malaria, including developmental delays, cognitive impairment, post-malaria neurological syndrome, and anemia.

## Conclusions

Malaria remains a significant public health concern with a substantial impact on children’s HRQoL. The association between malaria vaccination and higher utility values underscores the dual benefits of immunization in reducing disease burden and preserving quality of life, thereby reinforcing the need for expanded vaccine coverage. The significant disparities across the three value sets emphasize the need for localized preference weights in economic evaluations.

## Data Availability

The datasets generated during and/or analyzed during the current study are available from the corresponding author on reasonable request.

## Abbreviations

DALYs: Disability-Adjusted Life Years
EQ-VT: EuroQol Valuation Technology
HRQoL: Health-related Quality of Life
HTA: Health Technology Assessment
MCID: Minimal Clinically Important Difference
MAUM: Multi-Attribute Utility Measure
NICE: National Institute for Health and Care Excellence
PBAC: Pharmaceutical Benefits Advisory Committee
QALYs: Quality-Adjusted Life Years
UHC: Universal Health Coverage
