# Correction: Determining health-related quality of life among children with malaria in Kenya: does the choice of EQ-5D value set matter?

**DOI:** 10.1186/s12955-026-02664-z

**Published:** 2026-11-03

**Authors:** Tabitha Okech, Montarat Thavorncharoensap, Usa Chaikledkaew, George Paul Omondi, Patricia Nyokabi, S. M. Thumbi, Ammarin Thakkinstian

**Affiliations:** 1 Mahidol University Health Technology Assessment (MUHTA) Graduate Program, Bangkok, Thailand; 2 Center for Epidemiological Modelling and Analysis, University of Nairobi, Nairobi, Kenya; 3 Department of Pharmacy, Faculty of Pharmacy, Mahidol University, Bangkok, Thailand; 4 Animal Health Innovation Lab, Department of Clinical Studies, University of Nairobi, Nairobi, Kenya; 5 Ministry of Health, Nairobi, Kenya; 6 Paul G. Allen School for Global Health, Washington State University, Washington, USA; 7 Institute of Immunology and Infection Research, University of Edinburgh, Edinburgh, UK; 8 Department of Clinical Epidemiology and Biostatistics, Faculty of Medicine Ramathibodi Hospital, Mahidol University, Bangkok, Thailand

**Correction: Health and Quality of Life Outcomes (2025) 23:114**

http://orcid.org/10.1186/s12955-025-02445-0

The Ethics statement in the original article should instead read as follows:

Ethics approval and consent to participate:

This study received authority transfer from Mahidol University Institutional Review Board-MU-DT/PY-IRB (78.0319/EC.0366) to Maseno University Scientific and Ethics Review Committee, Kenya (MUSERC/01321/23). It was also approved by the Kenya National Council for Science, Technology and Innovation (NACOSTI/P/24/35525).

